# Intra-Site Variability in the Still Bay Fauna at Blombos Cave: Implications for Explanatory Models of the Middle Stone Age Cultural and Technological Evolution

**DOI:** 10.1371/journal.pone.0144866

**Published:** 2015-12-14

**Authors:** Emmanuel Discamps, Christopher Stuart Henshilwood

**Affiliations:** 1 Institute for Archaeology, History, Culture and Religious Studies, University of Bergen, Øysteinsgate 3, Bergen, Norway; 2 Université Bordeaux 1, CNRS UMR 5199 PACEA, Avenue des Facultés, Talence, France; 3 Evolutionary Studies Institute, University of the Witwatersrand, 1 Jan Smuts Avenue, Braamfontein 2000, Johannesburg, South Africa; University of Oxford, UNITED KINGDOM

## Abstract

To explain cultural and technological innovations in the Middle Stone Age (MSA) of southern Africa, scholars invoke several factors. A major question in this research theme is whether MSA technocomplexes are adapted to a particular set of environmental conditions and subsistence strategies or, on the contrary, to a wide range of different foraging behaviours. While faunal studies provide key information for addressing these factors, most analyses do not assess intra-technocomplex variability of faunal exploitation (*i*.*e*. variability *within* MSA phases). In this study, we assess the spatial variability of the Still Bay fauna in one phase (M1) of the Blombos Cave sequence. Analyses of taxonomic composition, taphonomic alterations and combustion patterns reveal important faunal variability both across space (lateral variation in the post-depositional history of the deposits, spatial organisation of combustion features) and over time (fine-scale diachronic changes throughout a single phase). Our results show how grouping material prior to zooarchaeological interpretations (*e*.*g*. by layer or phase) can induce a loss of information. Finally, we discuss how multiple independent subdivisions of archaeological sequences can improve our understanding of both the timing of different changes (for example in technology, culture, subsistence, environment) and how they may be inter-related.

## Introduction

In southern Africa, many scholars have attempted to explain the emergence and subsequent disappearance of cultural and technological innovations typical of the Middle Stone Age (MSA). Explanatory models have moved beyond single factor hypotheses and now often invoke the interplay between technology, culture, social dynamics, historical factors, demography, environment, land-use or subsistence strategies (*e*.*g*. [1–15]). In these avenues of research, faunal studies play an important role by providing key information on how MSA groups adapted their technology and subsistence strategies to changes in their local environment and the availability of prey. Recent work has highlighted variability in faunal exploitation patterns throughout the different MSA cultural phases (*e*.*g*. [11,16–18]), overturning the long-held perception of somewhat stable MSA subsistence strategies. This opened up the possibility that several MSA technological innovations may be partially linked to changes in foraging behaviour. Yet, if differences in subsistence strategies have been identified between MSA technocomplexes, such as the Still Bay and the Howiesons Poort, finer-scale studies focusing on intra-technocomplex variability (*i*.*e*. *within* MSA phases) are still rare. Theoretical frameworks built on ethnoarchaeological studies predict that a particular set of technologies should be capable of accommodating a wide range of foraging behaviours, depending on, for example, site function or local environmental conditions [4]. It follows that each MSA technocomplex was potentially adapted to a range of environmental conditions and involved different subsistence strategies. The question remains to what extent each technocomplex was flexible in this respect. Establishing whether inter- or intra-technocomplex variability is more important is thus essential in order to properly assess associations between certain technological innovations and particular subsistence strategies or environments, as well as their potential evolutionary links.

Recent zooarchaeological analyses at Klipdrift Shelter and Sibudu have shown a considerable degree of variability in faunal exploitation throughout, respectively, the Howiesons Poort [19,20] and post-Howiesons Poort [21]. The goal of the present study is to test if intra-technocomplex variability can be observed in the faunal signal of the Still Bay, another well-known technocomplex of the South African MSA, at Blombos Cave. Intra-technocomplex variability in subsistence strategies might express itself in two dimensions: over time (diachronic changes within a cultural phase) or across space (according to the site function at the landscape scale or, at the site scale, when processing activities are spatially organised). Distinguishing the two can prove challenging in the fossil record. At the site scale, the simultaneous study of lateral and diachronic variability allows stratigraphic patterns pertaining to genuine changes in subsistence strategies to be discerned from those due to random sampling of laterally variable behaviours. By exploring fine-scale intra-site variability, this study underlines broader implications for when faunal analysts should (or shouldn’t) group material from different squares or layers prior to zooarchaeological interpretations.

## Material and Methods

### Site background and selected sample

**Fig 1:**
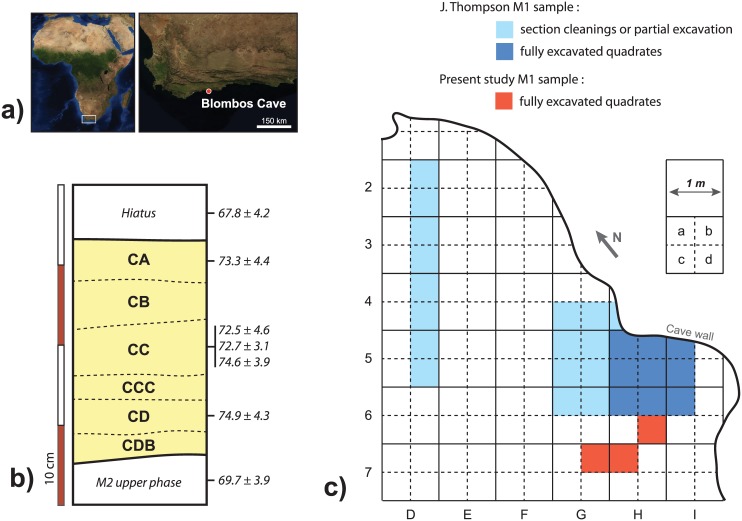
Blombos Cave location (a), simplified stratigraphy (b, with sub-divisions of the M1 phase and absolute dates from [23] in ka cal. BP) and site map (c, with quadrates analysed by J. Thompson and in this study). Satellite maps: NASA Worldview, public domain.

Blombos Cave, hereafter BBC, located on the southern coast of South Africa about 300 km east of Cape Town (Fig 1a), is one of the few archaeological sites that has well-preserved faunal remains associated with the Still Bay technocomplex [22]. The MSA deposits are divided in four major phases, from top to bottom: M1, M2 upper, M2 lower, M3. In order to test if certain characteristics of the faunal material varied within one of these major phases, this study focuses on the fauna from phase M1 (ca. 73–75±4 ka cal. BP, Fig 1b, [23]) attributed to the Still Bay.

Previous zooarchaeological analyses of the BBC Still Bay fauna grouped material according to the major BBC phases and thus provide no information on subsistence variability within, for example, the M1 phase ([22] on material from the 1992–1999 excavations; or [24–27] on material from the 2000–2004 excavations). However, each phase is composed of several layers distinguished during excavation based on composition, colour or texture [22]. The site is divided into 50 x 50 cm “quadrates” (sub-square units), each of which was hand excavated according to the stratigraphy. After measuring the total volume of buckets by quadrate and layer, non-plotted material was sieved through 3 and 1.5 mm nested sieves and retained for analysis. In 2011, the topographic surfaces of stratigraphic layers were recorded in each quadrate using the 3D scanning function of a Trimble VX Total Station (ca. 500 points per scan). This allows for detailed *a posteriori* reconstruction of the surface of each layer, even if quadrates were not necessarily excavated simultaneously.

For this study, the M1 macro-faunal material from three quadrates excavated in 2011 (G7b, H6d and H7a, Fig 1c) was analysed. In these quadrates, the M1 phase is about 25 to 30 cm thick and divided in 6 main layers (CA to CDB, from top to bottom) of around 3 to 5 cm each (Fig 1b). This material includes 32 plotted bones in addition to several thousand remains recovered during sieving (Table 1). Shellfish, fish, micro-mammals (*i*.*e*. smaller than the Cape dune molerat), amphibians and bird remains are excluded. All necessary permits were obtained for the described study, which complied with all relevant regulations. Excavation permit 2011/09/001 was issued to CSH by Heritage Western Cape under section 35 (4) of the National Heritage Resources Act no 25. The material is housed and curated by the Iziko Museums of South Africa, Queen Victoria Street, Cape Town, and at the University of the Witwatersrand Satellite Laboratory, Buitenkant Street, Cape Town.

**Table 1:** Material analysed by quadrate and layer.

Layer	Quad.	Mammals ID (NSP)	MammalsNID >2cm (NSP)	Tortoise bones (NSP)	Tortoise shell fr. >2cm (NSP)	Tortoise shell fr. <2cm (g)	NID <2cm (g)
CA	G7b	25	56	26	43	54.86	97.95
	H6d	45	98	41	175	99.71	96.10
	H7a	29	50	46	54	80.74	66.95
CB	G7b	50	120	21	44	32.88	127.59
	H6d	3	7	2	7	4.57	16.45
	H7a	18	26	9	14	13.71	39.29
CC	G7b	47	92	30	20	86.29	192.13
	H6d	28	58	14	59	33.98	66.61
	H7a	52	157	40	68	85.96	204.31
CCC	G7b	19	31	25	26	62.82	62.03
	H6d	17	32	4	14	17.11	53.16
	H7a	32	39	24	34	68.27	61.14
CD	G7b	14	21	21	17	32.53	29.93
	H6d	92	156	61	220	171.15	194.65
	H7a	19	47	16	13	31.83	48.90
CDB	G7b	28	16	16	25	27.96	24.77
	H6d	10	6	10	7	7.71	19.99
	H7a	17	24	23	19	24.76	26.60
**Total**		545	1036	429	859	936.84	1428.55

Quad.: quadrate; NSP: number of specimens; ID: identified specimens; NID: unidentified specimens; fr.:fragments; g: mass in grams.

### Analytical methods

Three main aspects of the faunal material were investigated for variability: taxonomic composition, taphonomic alterations and combustion patterns. Variability is assessed first between quadrates of a given layer (lateral variability) and then across layers (diachronic changes). Taxonomic composition and combustion patterns provide direct information on subsistence strategies in terms of hunted prey and bone processing practices, respectively. Further, the analysis of taphonomic alterations can shed light on site formation processes and the post-depositional history of bone assemblages. Taphonomic processes can vary in time and space, and hence differently bias zooarchaeological interpretations. Taphonomic data thus has to be analysed and discussed in the same framework as other, more direct, proxies of subsistence strategies.

All pieces were identified as precisely as possible to skeletal element and species or size class (adapted from [28], Table 2). Faunal samples were however too small for evaluating potential differences in skeletal-part profiles between quadrates and layers (S1 Table). Unidentified mammal bones and tortoise shell fragments longer than 2 cm were counted, while those smaller than 2 cm were weighed (Table 1). Tortoise shell fragments were not included in the analysis of taxonomic composition: they are easy to identify, even when highly fragmented, and thus their inclusion would exaggerate the representation of tortoise [17].

**Table 2:** Size classes used for mammals, ungulates and bovids.

Small mammals (< 4.5 kg)	-	-	*e*.*g*. Cape dune molerat, Rock hyrax
Large mammals (> 4.5 kg)	Small ungulates	Bovid 1	*e*.*g*. Grysbok
		Bovid 2	*e*.*g*. Reedbuck
	Large ungulates	Bovid 3	*e*.*g*. Wildebeest
		Bovid 4	*e*.*g*. Buffalo
		Bovid 5	*e*.*g*. Giant buffalo

Adapted from [28], with examples in the last column.

Detailed data on taphonomic alterations was recorded for all identified mammal remains, all unidentified mammal remains larger than 4 cm and a sample of unidentified bone fragments smaller than 4 cm, resulting in a total sample of 796 specimens (including 40% of all the mammal bones larger than 2 cm). Taphonomic data was not recorded for tortoise remains (for details concerning tortoise taphonomy see [25]). Cortical surfaces were observed under low-angled light using a 30x hand lens and a stereomicroscope when necessary. All taphonomic alterations were recorded, either of natural (*e*.*g*. concretions, root etching, manganese deposits, weathering, chemical alteration, tooth and trampling marks) or anthropic origin (*e*.*g*. cut and percussion marks). The general colour of the bone was also coded, as well as the proportion of cortical surface per fragment that was sufficiently well preserved for taphonomic analysis (in 4 classes: 0–25%, 25–50%, 50–75% or 75–100%). Fracture patterns of long bones were recorded as recent, green or dry break following criteria developed by Villa and Mahieu [29].

To best compare proportions of taxonomic groups and taphonomic alterations between quadrates and layers while accommodating for sample size, we systematically computed adjusted Wald proportions and confidence intervals [30]. The graphical representation of proportions with 95% confidence intervals allows for quick and efficient comparison while taking into account biases due to small sample sizes. This approach was coupled with chi-square tests performed using the PAST software suite [31].

To investigate variability in the combustion patterns of bone fragments, a multivariate approach similar to the one described by Costamagno et al. [32] was performed. At BBC, manganese staining is mostly limited to small black spots and cortical surfaces are well preserved: burnt bones could thus be distinguished by their colour, texture and cracked surface. All mammal remains were classified by length, degree of burning (*i*.*e*. unburnt, partially burnt, mostly black, grey or white, after [32,33]) and tissue type (*e*.*g*. compact, spongy). This approach distinguishes three main categories of combustion patterns: non-fuel burning (when bones are not intentionally burnt, for example as a consequence of roasting), use of bone as fuel (when bones are specifically processed and thrown into a fire to be used as fuel) and cleaning/fuel (when bones are thrown into the fire during site maintenance activities and/or to be used as fuel). Unlike the Costamagno et al. protocol, fragments smaller than 2 cm were not counted but weighed. In order to estimate the corresponding specimen numbers (NSP), bones smaller than 2 cm from 65 coarse fraction bags were both counted and weighted. For these 65 bags, bone counts and weights are statistically correlated (r = 0.68; p<0.0001), and calculations performed in PAST allowed us to estimate a mean of 7 bones smaller than 2 cm per gram, with about 5.7 to 8 bones per gram in 95% of the cases (RMA linear correlation, with a set intercept of 0: slope of 7.07±0.46, bootstrap 95%: 5.7 to 8.0). These numbers were subsequently used in the NSP calculations. The 1428.55 grams of bones smaller than 2 cm thus correspond to between approximately 8 to 11 thousand remains (8,143 to 11,428 bones). As suggested by Costamagno et al. [32], we computed the proportion of burnt bones smaller than 2 cm, the proportion of burnt bones that are carbonized or calcined (*i*.*e*. black, white or grey) and the proportion of carbonized or calcined bones that are spongy. Discriminant analyses of these ratios were subsequently performed using the PAST software to classify the BBC faunal samples, using Costamagno et al.’s [32] reference assemblages as a training set.

## Results

### Taxonomic variability

**Fig 2:**
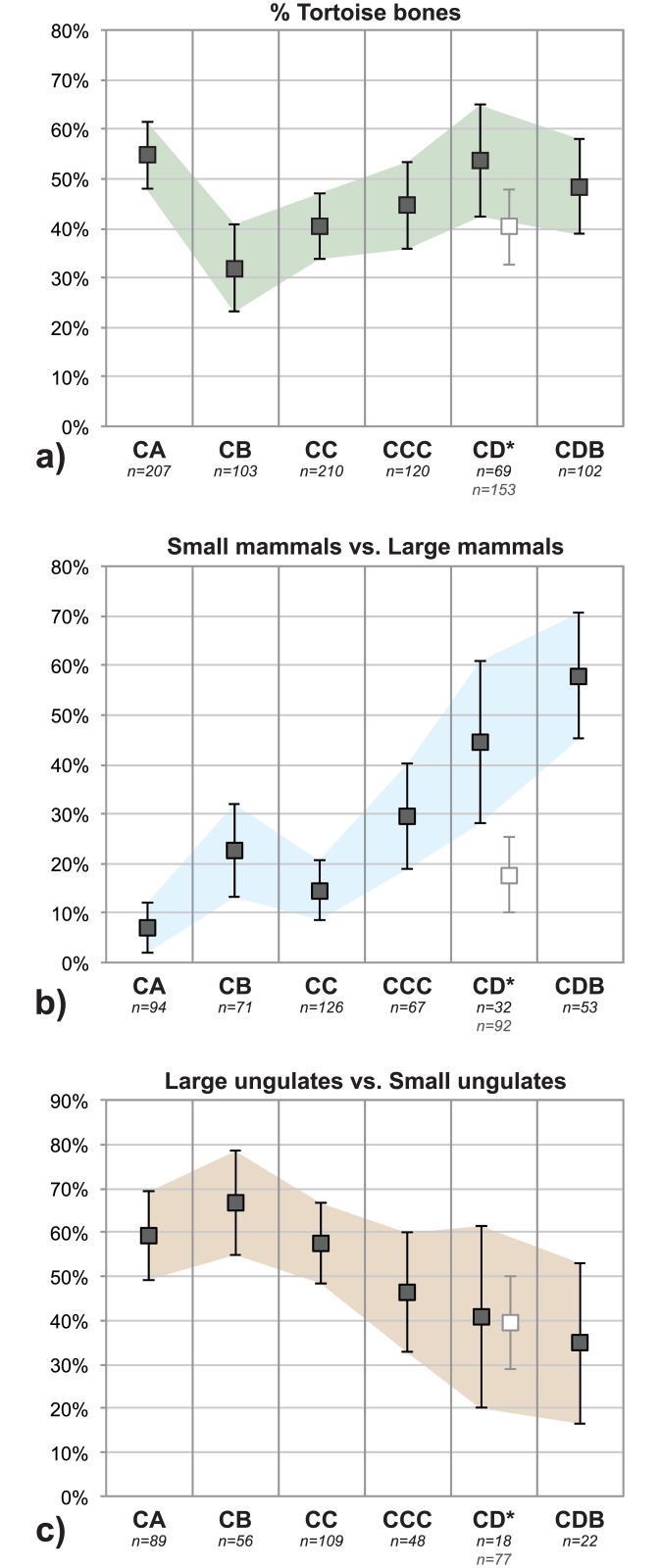
Adjusted proportions (with 95% confidence intervals) of main taxonomic groups. Material from all quadrates is grouped by layer except for CD (*: G7b+H7a in black symbols, H6d in white symbols).

Comparison of the main taxonomic groups allows for an initial assessment of differences in faunal composition between quadrates and layers. The relative abundance of tortoise bones, small mammals, small ungulates and large ungulates is not statistically different across quadrates G7b, H7a and H6d for layers CA, CB, CC, CCC and CDB (p>0.05 for all chi2 tests). Conversely, the proportions in quadrate H6d are notably different for layer CD (G7b vs. H6d: chi2 = 20.291; p<0.001). This is mainly due to a lower percentage of small mammals in H6d compared to other quadrates (Fig 2).

Aside from this lateral variability, three important diachronic changes are evident throughout the M1 phase (Fig 2, Table 3): 1) tortoise bones are less abundant in CB, 2) mammals are dominated by small species (*e*.*g*. molerat, hyrax) in CDB while these are rarer at the top of the sequence (CA-CC), and 3) large ungulates (*e*.*g*. eland, buffalo), uncommon in layers CCC-CDB, increase in proportion at the top of the sequence (CA-CC). Finer distinctions also exist: among small ungulates, size 2 bovids are more abundant that size 1 bovids in CB and CC, but not in other layers (Table 3).

**Table 3:** Numbers of identified specimens per layer.

	CA	CB	CC	CCC	CD (G7b & H7a)	CD (H6d)	CDB	Total
**Tortoise (bones only)**	113	32	84	53	37	61	49	429
**Cape dune molerat**		7	6	11	4	6	11	45
**Rock hyrax**		5		3	3	2	8	21
**Hare**	1		1					2
**Cape fur seal**	1			1				2
**Grysbok or steenbok**	7	1	4	6	1	3	2	24
**Reedbuck**			1	1		5		7
**Wildebeest or hartebeest**				1				1
**Eland**	5	4	8	1	1	3	1	23
**Buffalo**	1		3	1			1	6
**Small mammal indet.**	4	3	10	5	7	7	12	48
**Bovid indet.**	4		1		1		1	7
**Bovid 1**	19	5	12	6	7	20	9	78
**Bovid 1/2**	2	1	3	3		2	1	12
**Bovid 2**	8	11	26	10	3	17	3	78
**Bovid 2/3**							1	1
**Bovid 3**	3		1	1		1	1	7
**Bovid 3/4**	2		3	2	4	3	1	15
**Bovid 4**	2	2	8	1	1	6	1	21
**Large ungulate indet.**	40	32	40	15	1	17	2	147
**Total**	212	103	211	121	70	153	104	974

### Taphonomic variability

**Fig 3:**
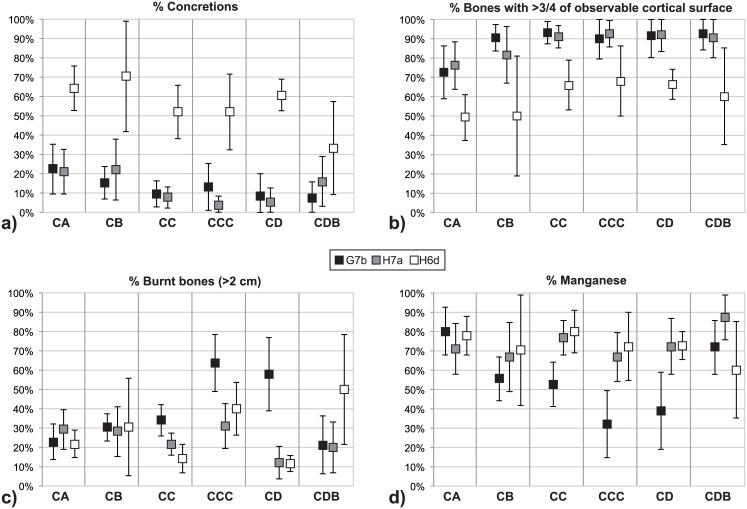
Lateral variability in taphonomic alterations. Adjusted proportions (with 95% confidence intervals) of concretions (a), well-preserved bones (b), burnt bones (c) and manganese staining (d).

Overall, the BBC M1 fauna is well preserved (Fig 3b). The most frequent post-depositional alterations are manganese deposits in the form of black spots (69.5%), concretions (26.5%), root etching (19.5%) and trampling marks (14%). Weathering is not uncommon (11%) but limited to the first stage (as defined by [34]), except in 8 cases (1% of the bones). Chemical alteration is rare (4%) and limited to slight alteration, except for one corroded bone.

**Fig 4:**
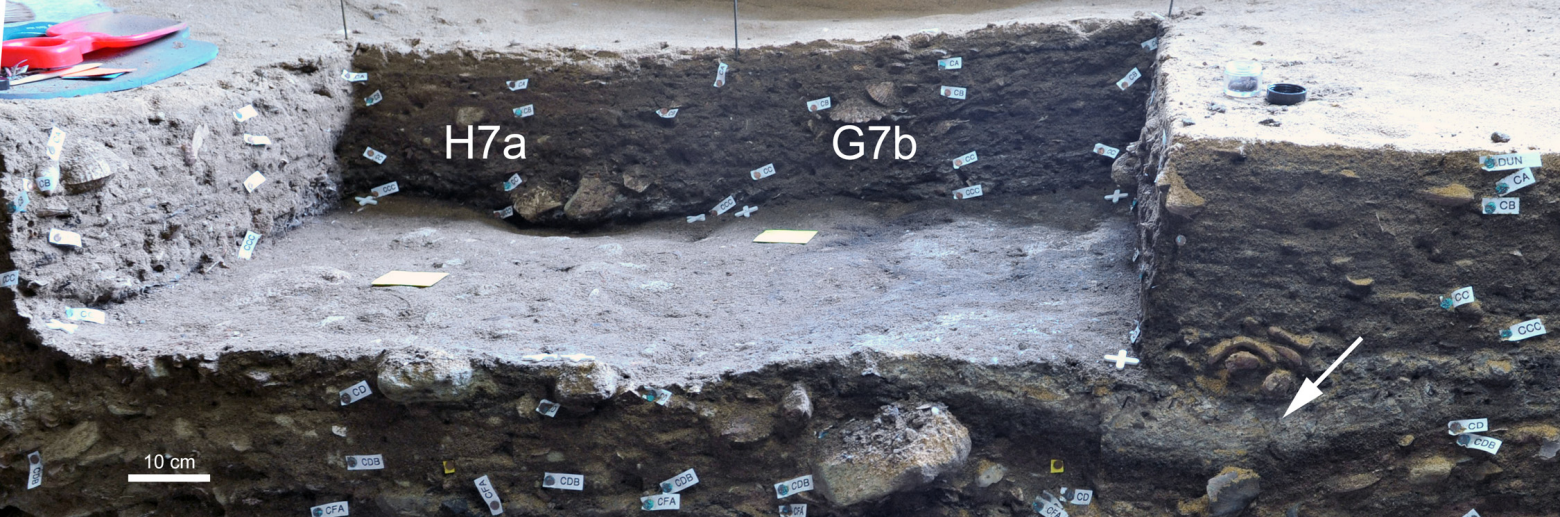
Surface of layer CD before excavation. Hearth features are apparent in quadrate G7b (on the right, white arrow) but not in quadrate H7a (left).

The relative importance of taphonomical alterations does not vary according to quadrate (*e*.*g*. for weathering, chemical alteration, root and trampling marks) apart from a few exceptions (Fig 3):

- In G7b and H7a, about 90% of the bones have more than 75% of their cortical surface well preserved (except for layer CA), while in H6d only 50 to 70% of the bones are equally well preserved. This pattern correlates with the frequency of concretions, which are about three times more abundant in H6d (50 to 70% in layers CA to CD) compared to G7b and H7a (5 to 20% in most layers). This is likely due to quadrate H6d being closer to the cave wall and the seepage of limestone rich water. When bones with concretions are excluded, 95% of H6d bones have more than 75% of their cortical surfaces well preserved. The same is true for G7b CA and H7a CA, going from about 75% of well-preserved cortical surfaces to 85% when concretions are excluded.
- In layers CC, CCC and CD, quadrate G7b has less evidence of manganese staining and, at the same time, a higher proportion of burnt bones. These two parameters are connected: black manganese dots on carbonized cortical surfaces are less easy to spot, thus explaining the inverse correlation between these taphonomical alterations. Large ashy features were recognized during excavation in G7b but not in H7a (Fig 4). Significant differences in the proportions of burnt bones between quadrates confirm that hearth features in the cave are spatially patterned [35].

Fig 3 shows how the taphonomic parameters mentioned above are interconnected, and confirms the existence of spatial patterning of taphonomic alterations. Concretions are significantly more abundant in H6d for layers CA to CD (and, consequently, well-preserved cortical surfaces are less frequent), as are burnt bones in G7b for layers CC to CD (as a result, the recorded proportion of manganese deposits is lower).

**Fig 5:**
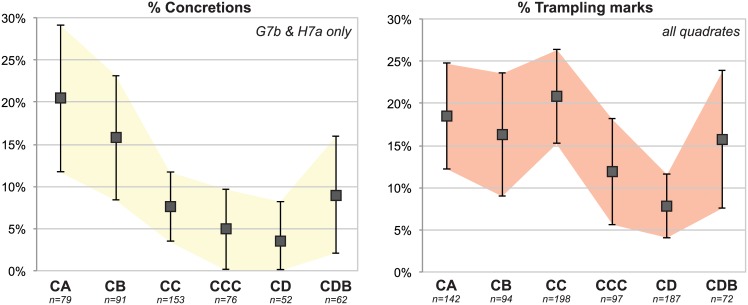
Diachronic variability in taphonomic alterations. Adjusted proportions (with 95% confidence intervals) of concretions in G7b and H7a (left), and trampling marks (right).

In addition to these lateral spatial variations, taphonomic alterations are also not evenly distributed across layers. While the abundance of trampling marks does not vary by quadrate, they are slightly more frequent in the top layers (CA to CC), even though the difference is significant only between layers CC and CD (Fig 5). In G7b and H7a, concretions are more abundant in the top layers as well (cf. discussion above on the preservation of cortical surfaces, Fig 5). In H7a and H6d, burnt bones larger than 2 cm are rarer in CD, while in G7b they are better represented in CCC and CD (Fig 3c).

**Fig 6:**
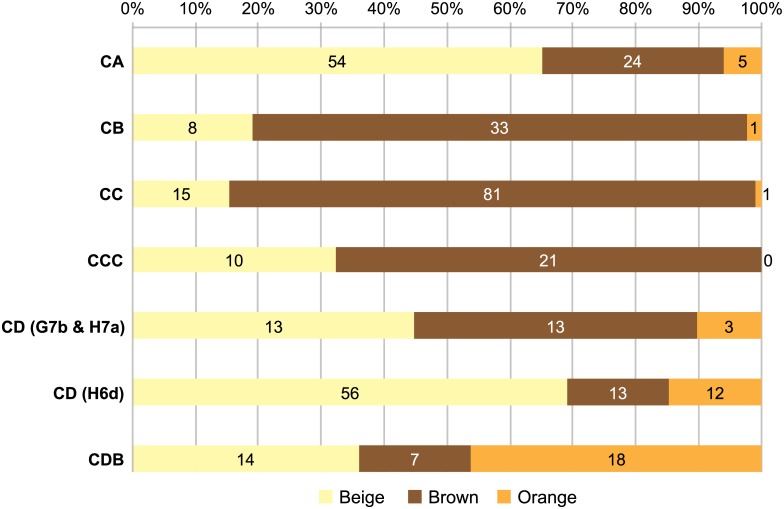
Proportions of major bone colour categories by layer. Burnt fragments are excluded and only the three most easily distinguishable colours are included (for example, bones recorded as “brown—orange” are excluded). Numbers show NSP values per category.

Bone colour also differs according to stratigraphic layers: “beige” bones are proportionally more abundant in CA, “brown” ones in CB to CCC, while CDB has a significant fraction of “orange” bones (Fig 6). While the factors underlying these colour variations are difficult to pinpoint, they may result from differences in the post-depositional history of faunal elements across layers.

Evidence of human exploitation on animal bones is ample, in the form of cut and percussion marks (Table 4) and most notably burnt bones (20 to 50% of all remains, Table 5). The abundance of cut and percussion marks does not significantly vary according to quadrate or layer. Bones identified as belonging to smaller or larger ungulates commonly have cut and percussion marks, and a large proportion of these bones are burnt (Table 4). Evidence of anthropogenic action on ungulate bones is present in all layers. In addition, only a small fraction of them were found complete (mostly carpotarsals, sesamoids and third phalanges), and fracture patterns support anthropogenic fracturing (92% of the ancient fractures that could be determined were made on green bone). On the contrary, only one bone bears carnivore marks, two have rodent marks, and one bone shows potential signs of digestion. This suggests very limited carnivore action and that the majority of ungulates in the analysed quadrates were brought to the site by MSA people. The carnivore contribution identified in our sample is lower than that identified by Thompson and Henshilwood [24], and may be connected to lateral variability in the depositional history of BBC M1 bones (as shown by Thompson and Henshilwood [24] in their Fig 7, p. 761).

**Fig 7:**
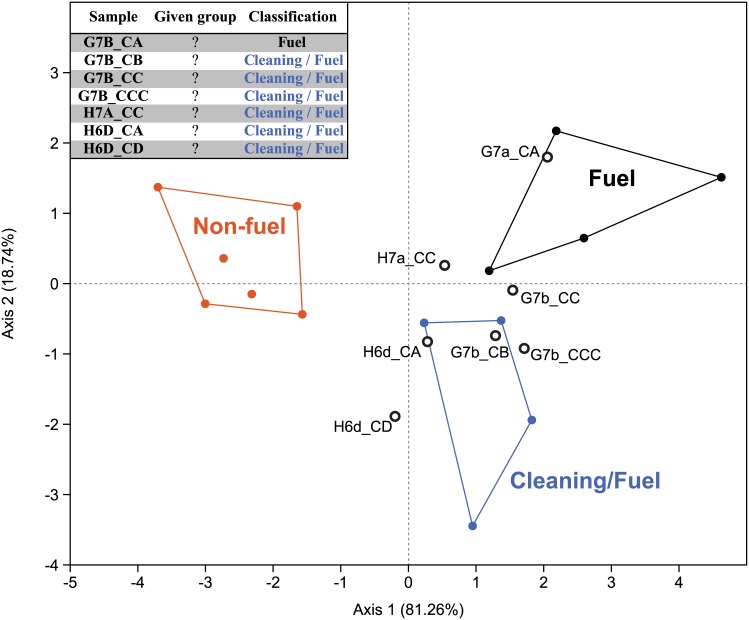
Discriminant analysis of burnt bones. Reference assemblages from Costamagno et al. [32] were used as a training set (plain symbols). The table in the top-left shows the classification of the selected BBC samples by the discriminant analysis (“?” in the given group column refers to the fact that the BBC samples were not used in the training set and only classified *a posteriori* by the discriminant analysis).

**Table 4:** Taphonomic data on agent of accumulation for identified specimens.

	Cut marks	Percussion marks	Complete bones	Carnivore/raptor modifications	Burnt bones
Small ungulates	7.2% (14)	4.5% (9)	6% (12)	TM: 0.5% (1); DIG: 0.5% (1)	29.5% (57)
Large ungulates	13% (28)	6.8% (15)	3.2% (7)	TM: 0% (0); DIG: 0% (0)	25.2% (55)
Small mammals	1.7% (2)	0% (0)	17.2% (20)	TM: 0% (0); DIG: 0% (0)	15.2% (17)

%NISP and NISP affected in brackets.

TM: tooth marks; DIG: digested remains.

**Table 5:** Characteristics of burnt bones.

Layer	Quadrate	Total burnt (estimated NSP)	Total burnt (%)	Burnt <2cm / Total burnt	Carbo. / Total burnt	Spongy / Carbo.
**CA**	**G7b**	**264–363**	**41–42%**	**93–95%**	**78–79%**	**52%**
	*H7a*	*186–252*	*40–41%*	*87–90%*	*61–62%*	*34%*
	**H6d**	**226–303**	**33%**	**84–88%**	**79–81%**	**17%**
**CB**	**G7b**	**294–391**	**33%**	**82–86%**	**87–88%**	**27%**
	*H7a*	*115–155*	*43%*	*87–90%*	*61–62%*	*29–30%*
	*H6d*	*26–36*	*25%*	*92–94%*	*79%*	*8–9%*
**CC**	**G7b**	**473–644**	**38%**	**89–92%**	**86%**	**31%**
	**H7a**	**441–600**	**32%**	**89–92%**	**76%**	**27%**
	*H6d*	*120–163*	*26%*	*88–91%*	*78–80%*	*27–28%*
**CCC**	**G7b**	**203–274**	**50%**	**86–89%**	**92%**	**26–27%**
	*H7a*	*173–232*	*41%*	*85–89%*	*80–81%*	*27–28%*
	*H6d*	*111–149*	*31–32%*	*83–87%*	*71–73%*	*0%*
**CD**	*G7b*	*110–148*	*54%*	*85–89%*	*92%*	*34–35%*
	*H7a*	*50–68*	*14–15%*	*90–93%*	*93–94%*	*21–22%*
	**H6d**	**268–362**	**20%**	**87–91%**	**82–83%**	**1–2%**
**CDB**	*G7b*	*25–33*	*14%*	*76–82%*	*74–75%*	*24%*
	*H7a*	*21–27*	*10–11%*	*71–78%*	*69–72%*	*10%*
	*H6d*	*8–9*	*5–6%*	*39–47%*	*100%*	*21–25%*

Italic characters correspond to samples of small size that are not discussed in the text and not included in the discriminant analysis. Carbo.: carbonized or calcined.

The status of small mammals is more equivocal. Even though 15% of small mammal bones are burnt (similar burning patterns are present in all layers except CDB), this data is hard to interpret in terms of the agents responsible for their accumulation, as the burning of these small bones might be accidental. One molerat incisor however shows a partial burning pattern consistent with roasting on an open-fire [36]. Direct evidence of human processing is rare (2 bones with cut marks, in layers CD and CDB), and evidence of raptor or carnivore modification is absent (Table 4). The scarcity of cut marks is to be expected in the case of an anthropogenic accumulation, as little or no butchering is required for humans to consume small mammal carcasses [37–39]. Only 17% of small mammal bones were found complete (mostly metapodials and phalanges), and articulated elements were not noted during excavation. Post-cranial elements (NISP = 81) outnumber cranial ones (NISP = 15, excluding 20 isolated teeth, S1 Table). Despite the fact that the interpretation of small mammal accumulation is often ambiguous, this data supports an anthropogenic rather than raptor, carnivore or natural accumulation (see [39] for a recent synthesis on this issue).

### Combustion patterns

Table 5 presents the data acquired on burnt bones per quadrate and layer following Costamagno et al. [32] protocol. Variability in combustion patterns is evident across both quadrates and layers, but is probably partially linked to small sample sizes (as for example with layer CDB). Thus, only samples with a minimum total NSP estimate of more than 200 remains are discussed below. In all these cases (Table 5), burnt bones are dominated by fragments smaller than 2 cm (>80%) and most are carbonized or calcined (>75%). Calcined bones are present in all layers and quadrates, often representing more than 30% of the burnt bones (and up to 57% of them in G7b CCC). According to Stiner et al. [33], bones have to be in direct contact with fire to be calcined. Their abundance at BBC would thus preclude accidental burning from overlying hearths as a main cause of burning.

Results of the discriminant analysis are presented in Fig 7: most quadrates and layers can be assigned to the “cleaning / fuel” category. Bones were thus presumably not burnt accidently, but rather thrown into fireplaces as a part of cleaning the living space. This behaviour would be similar to that proposed for Sibudu [40,41], where bones were likely burnt as a consequence of site maintenance activities (disposal of food waste in fire or repetitive and intentional burning of bedding).

The CA sample from quadrate G7b is different due to a higher percentage of spongy bones in the carbonized fraction (52%). In contrast, for the same layer (CA) in adjacent quadrate H7a, spongy portions are scarcer in the carbonized fraction (34%) and are found in quantities comparable to those in the unburnt fraction (29%). G7b CA is classified as “fuel” by discriminant analysis: this particular sample potentially reflects a distinctive behaviour where spongy portions were preferentially selected and thrown into the fire and consumed as fuel. These patterns need further investigation before the use of bone as fuel in BBC can be confirmed, notably through experimentation. It remains possible that the Costamagno et al. [32] reference sample, set for Palaeolithic Europe, is not adapted to MSA contexts where prey species differ.

## Discussion

The BBC M1 faunal assemblage presents examples of both lateral and diachronic variability. As a consequence of small sample sizes, faunal analysts are often forced to group material from different squares or different layers, with the assumption that they all relate to the same faunal assemblage (*e*.*g*. [24,27,42]). The BBC M1 material analysed here provides an instructive example of why spatial variability should be more carefully assessed before excavation units are combined. This is all the more striking as our analyses show examples of lateral variability at a small scale, between sub-squares. In the following, we highlight examples of faunal variability arising from different causes, and why considering this variability is valuable.

### Lateral variability: difficulties encountered in the field?

**Fig 8:**
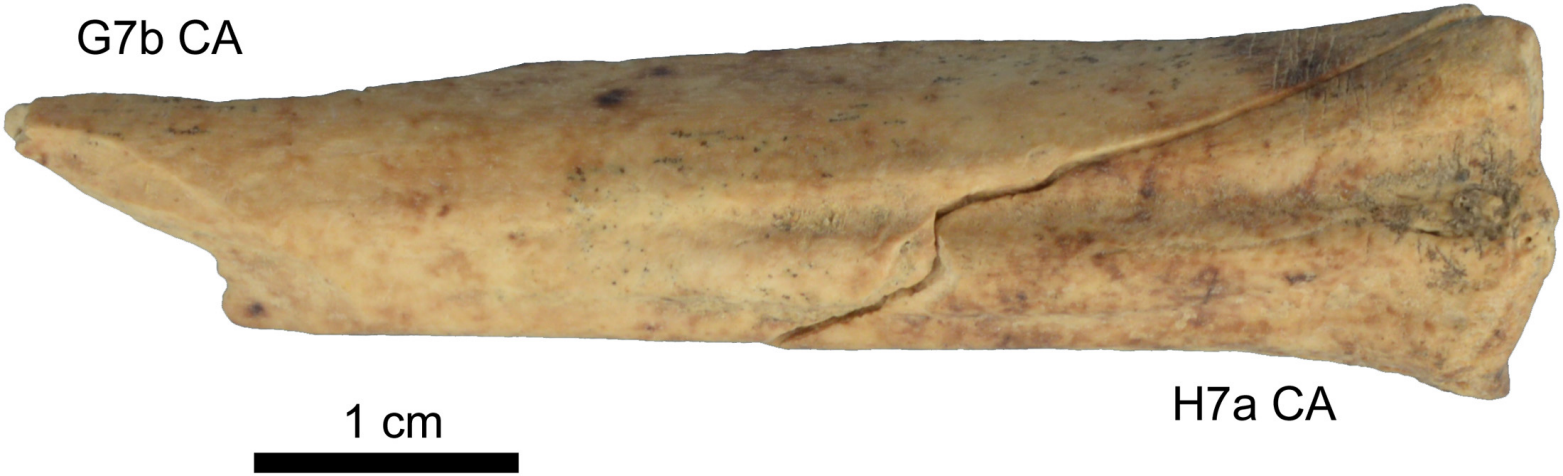
Refit between fragments collected in two different quadrates and assigned to the same layer (*Raphicerus sp*. metatarsal).

When examined layer by layer, the taxonomic composition and the frequency of most taphonomic alterations do not vary significantly by quadrate (except for layer CD, as discussed below). As such, faunal material collected in different quadrates can be grouped without losing information, as is often done in faunal analyses. In order to further test the stratigraphic integrity of BBC M1 layers, refitting between faunal fragments was attempted. Only three refits were found on ancient breaks, two of which involved fragments from the same layer and quadrate, thus providing limited information. A third refit, between two fragments from H7a CA and G7b CA (Fig 8), supports combining the CA material from these two quadrates.

**Fig 9:**
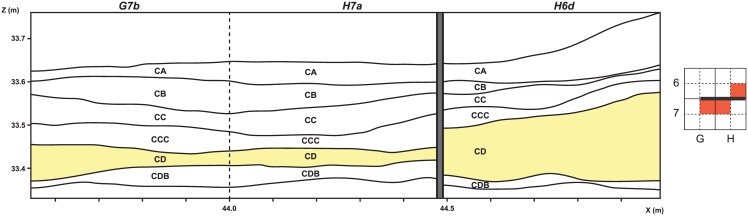
Virtual cross-section between trenches 6 and 7 (left, layer CD is shown in beige), next to a site plan with the three quadrates concerned in red (right). Limits between layers were estimated from 3D surface scan points.

The situation is more subtle for layer CD. In this layer, the taxonomic composition of H6d is statistically different from other quadrates. Although these quadrates were not excavated at the same time, surface scans acquired during fieldwork allow us to reconstruct a “virtual” cross-section (Fig 9). While the delimitations of most layers seem in good agreement across the three quadrates, layer CD is several centimetres thicker in H6d, such that material labelled “H6d CD” potentially includes some material from layers CCC and CDB (as defined in other quadrates). Inspection of excavation records and photographs reveals the presence of large calcrete blocks in H6d in layers CCC and CD, rendering the distinction of layers difficult in the field. This may explain why the faunal composition of H6d CD differs from other quadrates. Without detailed analysis of spatial patterning and collaborative work with the principal excavators, such problems might have remained unseen.

### Lateral variability: differences in depositional and/or post-depositional history?

Lateral spatial patterning of taphonomic alterations has been previously described for faunal assemblages in other sedimentary contexts (*e*.*g*. [43,44]), and BBC M1 adds to this record. The necessity of taphonomic analyses is often highlighted in MSA zooarchaeological research (*e*.*g*. [18,45]), yet the benefits of analysing this type of data spatially is seldom recognised. Significant lateral variation is evident in the abundance of concretions in BBC, and the preservation of cortical surfaces differs between quadrates. Spatial variability in the post-depositional history of the deposits might, in itself, induce differences in observed cut-mark frequencies. Even if a spatial approach to taphonomic alterations does not provide significant information on past human behaviour, it nevertheless allows for better discernment of the limits imposed by depositional and post-depositional processes on zooarchaeological interpretations.

### Lateral variability: subtle differences in human behaviour? The challenge imposed by deposits with well-preserved structures

BBC is one of the few MSA sites in South Africa where depositional conditions allowed for the preservation of *in situ* anthropogenic structures such as hearths and other features [46]. In BBC M1, spatial variability is apparent in the characteristics of burnt bones. Differences in combustion patterns are probably a by-product of spatially differentiated behaviours in the past. The within-site spatial distribution of hearths affected the percentage of burnt bones per quadrate, but, moreover, potential differences in the functional use of these hearths is also perceptible from analyses of burnt bones. This likelihood is seldom considered before material from different quadrates is combined and analysed as one faunal assemblage. Somewhat counter-intuitively, an excellent degree of preservation presents challenges to traditional zooarchaeological analyses, in the sense that faunal material from adjacent squares from the same layer cannot be considered as a palimpsest, and thus should not be studied as a whole. For example, differences between G7b CA combustion patterns and others quadrates from the same layer would not have been detected if the material had been studied by layer. Grouped with other combustion features, a potential distinct behaviour (the use of bone as fuel) would have remained unseen. Larger samples are therefore not always better, as they can mask some behavioural signatures in the case of archaeological sites with good spatial integrity. Future research at BBC, such as micro-morphological analyses of ashy features and distributional analysis of burnt lithics, should complement faunal data, producing a better understanding of lateral variability in MSA fire-making behaviour.

### Fine-scale diachronic variability

**Fig 10:**
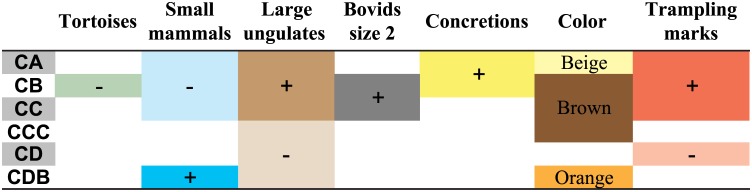
Summary of diachronic patterns perceived in BBC M1 phase.

The BBC fauna shows chronological changes in both taxonomic composition and taphonomic alterations within a single phase (Fig 10). The top layers of phase M1 (CA-CC) differ from the underlying ones (CCC-CDB) in that they have higher proportions of large ungulates and small mammals are less frequent. Taphonomic variation in bone preservation (concretions, trampling marks, colour) is also observable. That these parameters change in a similar way throughout the stratigraphy in different quadrates, while other factors can be shown to vary laterally (cf. above), argues in favour of genuine diachronic changes. In contrast, stratigraphic patterns in the abundance of burnt bones should not be interpreted as evidence for varying intensity of fire use through time, as these patterns may be only due to the random sampling of hearth features, the positions of which varied in the cave over time. Pending detailed geoarchaeological data on site formation processes and the realisation of other refitting programs (*e*.*g*. on lithic artefacts), discussing the stratigraphic integrity of the BBC M1 layers is still limited. Nevertheless, the identification of these fine-scale chronological patterns supports the hypothesis that the BBC M1 layers record different occupational phases.

**Fig 11:**
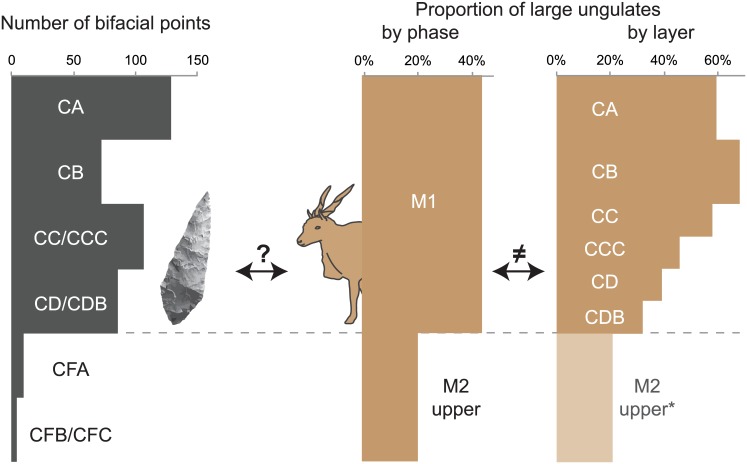
Comparison between the number of Still Bay points (left, data from [35,51]) and the proportion of large ungulates (right, in %NISP of the total number of ungulates). The marked increase in the frequency of Still Bay points between phases M2 upper and M1 seems correlated with a faunal shift when data is analysed by phase (data from [24]), while the latter appears much more gradual if the data is considered by layer (*: data from present study for M1 layers and from [24] for M2 upper). Note that the datasets compared here do not come from the same quadrates (all BBC quadrates for Still Bay points, different samples for faunal remains cf. Fig 1c).

An increase in the proportion of large ungulates between phases M2 upper and M1 has been highlighted for the BBC sequence [22,24], but our results show this shift is gradual, with high proportions of large ungulates only near the top of the M1 phase, and not earlier. This has implications for the potential synchronicity of technological and subsistence changes. At BBC, the distinction between M2 upper and M1 phases is in part based on the increased abundance of Still Bay points in phase M1 (Fig 11). If faunal patterns are analysed according to phases, this technological change seems correlated with the increase in the proportion of large ungulates, but finer-scale analysis shows that this faunal change is more progressive than the shift in the frequency of bifacial points (Fig 11).

**Fig 12:**
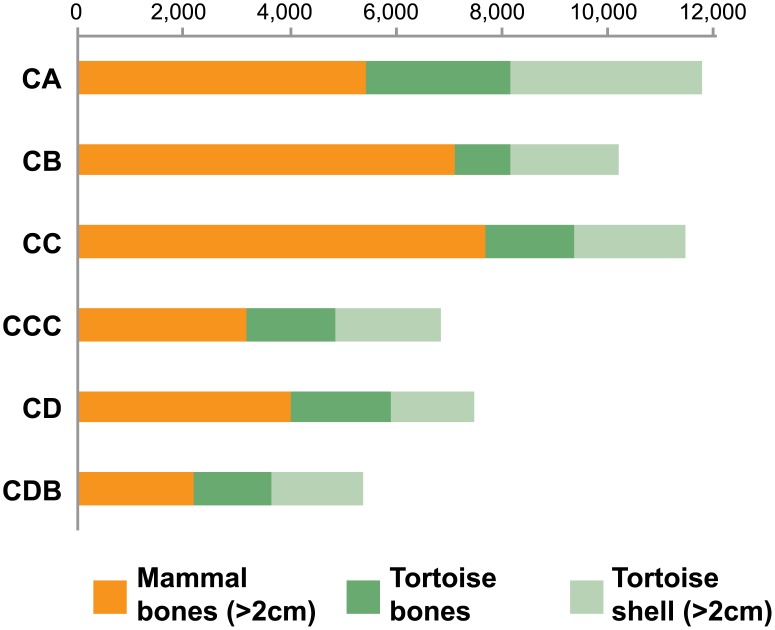
Density of terrestrial faunal remains. NSP per m^3^ of sediment, G7b and H7a quadrates only.

Diachronic changes within M1 have already been identified at BBC through differences in material culture [47,48] but not in the macro-faunal composition. Higher numbers of large ungulate grazers near the top of M1 might relate to environmental changes, with an increase of open grassland habitats in the vicinity of BBC during MIS 4 [49]. This taxonomic shift also corresponds to higher proportions of trampling marks, and a marked increase in the density of terrestrial faunal material. While density values vary slightly between quadrates, faunal fragments are generally more abundant in layers CA to CC (Fig 12). Lithic counts are not yet available for the quadrates analysed here, but previous analyses in other quadrates showed that the top layers of the M1 phase have twice the density of lithic artefacts compared to the bottom layers [22]. While sorting the faunal material, we also noted that micro-faunal remains were more abundant in layers CD and CDB (as noted by [49] in other quadrates). Taken together, this data (abundance of trampling marks, density of macro-, micro-faunal and lithic remains) could suggest that the intensity of human occupations increased towards the end of the M1 phase, but pending detailed geoarchaeological or radiometric data on depositional rates, such conclusions can only be considered as working hypotheses (cf. [50]).

## Conclusion

Our study highlights considerable variability in the Still Bay fauna at BBC, both between quadrates and across layers of a single phase (M1). These results show how the premature combination of faunal material (*e*.*g*. by layer or phase) could induce a loss of significant information. Grouping material early in an analysis allows one to more easily tackle sample size issues, however this process can hinder the identification of lateral spatial variability as well as fine-scale diachronic changes in subsistence strategies.

Lateral variability becomes a significant issue in contexts with good degree of preservation of small human-made structures such as hearths. The example of BBC demonstrates how part of the spatial variability is due to lateral variation in the depositional and post-depositional history of the assemblages, as would be expected in a cave system. The BBC M1 deposits also preserve the spatial organisation of processing activities in the cave. Without spatial analysis, the potential use of bone as fuel in one specific sub-square would not have been recognised. However, part of the behavioural information obtained through spatial analysis might relate to epiphenomena. When focusing on larger-scale questions, it is essential to assess the significance of behaviours identified at a finer scale. For example, the potential use of bone as fuel in BBC quadrate G7b should not be considered as a characteristic of Still Bay processing strategies. Although it potentially extends the range of known behaviours, it should not, by itself, redefine our ideas of typical BBC Still Bay combustion activities. In this regard, analytical choices are facilitated by sedimentary contexts with important post-depositional redistribution of faunal material, as zooarchaeologists then directly access “general” / “averaged” patterns.

In addition to lateral variability, we identified diachronic faunal changes within the M1 phase at BBC, some of which were previously estimated to be coincident with the transition between the M2 upper and M1 phases. This has important implications for explanatory models of MSA technological and cultural evolution. Exploring relationships between different factors can be impaired by differences in the resolution of the compared datasets (as experienced by [15] at Sibudu). As Thompson [18] highlights, a lack of variability/change in other analyses (such as material culture) should not hinder the identification of changes in other spheres such as subsistence strategies. To overcome these issues, faunal analysts can explore their data spatially (by squares, layers or spits, or, when they are sufficiently abundant, through analyses of plotted material) and define their own assemblages and subdivisions afterwards, by combining only the material that shares the same characteristics, or in which variability is not considered to be of interest (as in the case of small-scale lateral variability, cf. above). Ideally, faunal assemblages should be defined independently from other criteria, such as sedimentology or lithic technology. Such an approach allows faunal analysts to construct independent chronologies for changes in subsistence strategies and palaeoenvironmental conditions [44,52]. The arrhythmic tempos of MSA cultural and technological changes have previously been argued by Porraz et al. [12]. By establishing independent subdivisions of archaeological deposits on different criteria, archaeologists can more precisely test if the changes they see in different behavioural spheres are synchronous or not, and how they might relate to one another.

## Supporting Information

S1 Table Skeletal-part profiles (NISP) for small mammals, small and large ungulates by layer. (XLS)

## References

[1] Klein RG. Archeology and the evolution of human behavior. Evol Anthropol. 2000;9: 17–36.

[2] Shennan S. Demography and Cultural Innovation: a Model and its Implications for the Emergence of Modern Human Culture. Cambridge Archaeol J. 2001;11: 5–16.

[3] Henshilwood CS, Marean CW. The origin of modern human behavior. Curr Anthropol. 2003;44: 627–651. 14971366 10.1086/377665

[4] Bird DW, O’Connell JF. Behavioral Ecology and Archaeology. J Archaeol Res. 2006;14: 143–188. 10.1007/s10814-006-9003-6

[5] Mellars P. Why did modern human populations disperse from Africa ca. 60,000 years ago? A new model. Proc Natl Acad Sci. 2006;103: 9381–9386. 16772383 10.1073/pnas.0510792103 PMC1480416

[6] McCall GS. Behavioral ecological models of lithic technological change during the later Middle Stone Age of South Africa. J Archaeol Sci. 2007;34: 1738–1751. 10.1016/j.jas.2006.12.015

[7] Jacobs Z, Roberts RG, Galbraith RF, Deacon HJ, Grün R, Mackay A, et al Ages for the Middle Stone Age of southern Africa: Implications for human behavior and dispersal. Science. 2008;322: 733–735. 10.1126/science.1162219 18974351

[8] Dusseldorp GL. Tracking the influence of technological change on Middle Stone Age hunting strategies in South Africa. Quat Int. 2012;270: 70–79. 10.1016/j.quaint.2011.02.011

[9] Dusseldorp GL. Explaining the Howiesons Poort to post-Howiesons Poort transition: a review of demographic and foraging adaptation models. Azania Archaeol Res Africa. 2014; 1–37. 10.1080/0067270X.2014.937080

[10] McCall GS, Thomas JT. Still Bay and Howiesons Poort Foraging Strategies: Recent Research and Models of Culture Change. African Archaeol Rev. 2012;29: 7–50. 10.1007/s10437-012-9107-y

[11] Clark JL, Kandel AW. The Evolutionary Implications of Variation in Human Hunting Strategies and Diet Breadth during the Middle Stone Age of Southern Africa. Curr Anthropol. 2013;54: S269–S287. 10.1086/673386

[12] Porraz G, Parkington JE, Rigaud J-P, Miller CE, Poggenpoel C, Tribolo C, et al The MSA sequence of Diepkloof and the history of southern African Late Pleistocene populations. J Archaeol Sci. 2013;40: 3542–3552. 10.1016/j.jas.2013.02.024

[13] Ziegler M, Simon MH, Hall IR, Barker S, Stringer C, Zahn R. Development of Middle Stone Age innovation linked to rapid climate change. Nat Commun. 2013;4: 1905–1909. 10.1038/ncomms2897 23695699 PMC4354264

[14] Wurz S. Technological Trends in the Middle Stone Age of South Africa between MIS 7 and MIS 3. Curr Anthropol. 2013;54: S305–S319. 10.1086/673283

[15] Conard NJ, Will M. Examining the Causes and Consequences of Short-Term Behavioral Change during the Middle Stone Age at Sibudu, South Africa. PLoS ONE. 2015;10: e0130001 10.1371/journal.pone.0130001 26098694 PMC4476744

[16] Lombard M, Clark JL. Variability and Change in Middle Stone Age Hunting Behaviour: Aspects from the Lithic and Faunal Records In: Badenhorst S, Mitchell P, Driver JC, editors. Animals and People Archaeozoological Papers in Honour of Ina Plug. Archaeopre. BAR International Series 1849; 2008 pp. 46–56.

[17] Steele TE, Klein RG. Late Pleistocene Subsistence Strategies and Resource Intensification in Africa In: Hublin J-J, Richards MP, editors. The Evolution of Hominin Diets: Integrating Approaches to the Study of Palaeolithic Subsistence Vertebrate Paleobiology and Paleoanthropology. Springer Netherlands; 2009 pp. 113–126.

[18] Thompson JC. Variability in Middle Stone Age faunal exploitation and use of the physical and social landscapes in the southwestern Cape, South Africa In: Delagnes A, Conard NJ, editors. Settlement Dynamics of the Middle Paleolithic and Middle Stone Age III. Tübingen: Kerns Verlag; 2010 pp. 1–28.

[19] Henshilwood CS, van Niekerk KL, Wurz S, Delagnes A, Armitage SJ, Rifkin RF, et al Klipdrift Shelter, southern Cape, South Africa: preliminary report on the Howiesons Poort layers. J Archaeol Sci. 2014;45: 284–303. 10.1016/j.jas.2014.01.033

[20] Reynard JP, Discamps E, Badenhorst S, van Niekerk K, Henshilwood CS. Subsistence strategies in the southern Cape during the Howiesons Poort: Taphonomic and zooarchaeological analyses of Klipdrift Shelter, South Africa. Quat Int. 2015; in press. 10.1016/j.quaint.2015.07.041

[21] Clark JL. Exploring the Relationship Between Climate Change and the Decline of the Howieson’s Poort at Sibudu Cave (South Africa) In: Clark JL, Speth JD, editors. Zooarchaeology and Modern Human Origins: Human Hunting Behavior during the Later Pleistocene Vertebrate Paleobiology and Paleoanthropology. Dordrecht: Springer Netherlands; 2013 pp. 9–18

[22] Henshilwood CS, Sealy JC, Yates R, Cruz-Uribe K, Goldberg P, Grine FE, et al Blombos Cave, southern Cape, South Africa: preliminary report on the 1992–1999 excavations of the Middle Stone Age levels. J Archaeol Sci. 2001;28: 421–448. 10.1006/jasc.2000.0638

[23] Jacobs Z, Hayes EH, Roberts RG, Galbraith RF, Henshilwood CS. An improved OSL chronology for the Still Bay layers at Blombos Cave, South Africa: further tests of single-grain dating procedures and a re-evaluation of the timing of the Still Bay industry across southern Africa. J Archaeol Sci. 2013;40: 579–594. 10.1016/j.jas.2012.06.037

[24] Thompson JC, Henshilwood CS. Taphonomic analysis of the Middle Stone Age larger mammal faunal assemblage from Blombos Cave, southern Cape, South Africa. J Hum Evol. 2011;60: 746–767. 10.1016/j.jhevol.2011.01.013 21470662

[25] Thompson JC, Henshilwood CS. Tortoise taphonomy and tortoise butchery patterns at Blombos Cave, South Africa. J Archaeol Sci. 2014;41: 214–229. 10.1016/j.jas.2013.08.017

[26] Thompson JC, Henshilwood CS. Nutritional values of tortoises relative to ungulates from the Middle Stone Age levels at Blombos Cave, South Africa: implications for foraging and social behaviour. J Hum Evol. 2014;67: 33–47. 10.1016/j.jhevol.2013.09.010 24423785

[27] Reynard JP, Badenhorst S, Henshilwood CS. Inferring animal size from the unidentified long bones from the Middle Stone Age layers at Blombos Cave, South Africa. Ann Ditsong Natl Museum Nat Hist. 2014;4: 9–25.

[28] Brain CK. Some suggested procedures in the analysis of bone accumulations from southern African Quaternary sites. Ann Transvaal Museum. 1974;29: 1–8.

[29] Villa P, Mahieu E. Breakage patterns of human long bones. J Hum Evol. 1991;21: 27–48.

[30] Agresti A, Coull BA. Approximate Is Better than “Exact” for Interval Estimation of Binomial Proportions. Am Stat. 1998;52: 119–126. 10.2307/2685469

[31] Hammer Ø, Harper DAT, Ryan PD. PAST: Paleontological statistics software package for education and data analysis. Palaeontol Electron. 2001;4: 9.

[32] Costamagno S, Théry-Parisot I, Castel J-C, Brugal J-P. Combustible ou non? Analyse multifactorielle et modèles explicatifs sur des ossements brûlés paléolithiques In: Théry-Parisot I, Costamagno S, Henry A, editors. Gestion des combustibles au Paléolithique et au Mésolithique : nouveaux outils, nouvelles interprétations. Archaeopress; 2009 pp. 65–84.

[33] Stiner MC, Kuhn SL, Weiner S, Bar-Yosef O. Differential Burning, Recrystallization, and Fragmentation of Archaeological Bone. J Archaeol Sci. 1995;22: 223–237. 10.1006/jasc.1995.0024

[34] Behrensmeyer AK. Taphonomic and ecologic information from bone weathering. Paleobiology. 1978;4: 150–162.

[35] Haaland MM. Intra-site spatial analysis of the Still Bay units in Blombos Cave, South Africa. Master thesis, University of Bergen. 2012.

[36] Henshilwood CS. Identifying the Collector: Evidence for Human Processing of the Cape Dune Mole-Rat,Bathyergus suillus, from Blombos Cave, Southern Cape, South Africa. J Archaeol Sci. 1997;24: 659–662. 10.1006/jasc.1996.0148

[37] Lyman RL. Vertebrate Taphonomy. University Press, Cambridge 1994.

[38] Blasco R, Rosell J, Fernández Peris J, Arsuaga JL, Bermúdez de Castro JM, Carbonell E. Environmental availability, behavioural diversity and diet: a zooarchaeological approach from the TD10-1 sublevel of Gran Dolina (Sierra de Atapuerca, Burgos, Spain) and Bolomor Cave (Valencia, Spain). Quat Sci Rev. 2013;70, 124–144.

[39] Badenhorst S, van Niekerk KL, Henshilwood CS. Rock Hyraxes (Procavia capensis) from Middle Stone Age Levels at Blombos Cave, South Africa. African Archaeol Rev. 2014;31: 25–43. 10.1007/s10437-014-9154-7

[40] Cain CR. Using burned animal bone to look at Middle Stone Age occupation and behavior. J Archaeol Sci. 2005;32: 873–884. 10.1016/j.jas.2005.01.005

[41] Clark JL, Ligouis B. Burned bone in the Howieson’s Poort and post-Howieson’s Poort Middle Stone Age deposits at Sibudu (South Africa): behavioral and taphonomic implications. J Archaeol Sci. 2010;37: 2650–2661. 10.1016/j.jas.2010.06.001

[42] Clark JL, Plug I. Animal exploitation strategies during the South African Middle Stone Age: Howiesons Poort and post-Howiesons Poort fauna from Sibudu Cave. J Hum Evol. 2008;54: 886–898. 10.1016/j.jhevol.2007.12.004 18234284

[43] Stiner MC, Kuhn SL, Surovell TA, Goldberg P, Meignen L, Weiner S, et al Bone Preservation in Hayonim Cave (Israel): a Macroscopic and Mineralogical Study. J Archaeol Sci. 2001;28: 643–659. 10.1006/jasc.2000.0634

[44] Discamps E, Delagnes A, Lenoir M, Tournepiche J-F. Human and Hyena Co-occurrences in Pleistocene sites : Insights from Spatial, Faunal and Lithic Analyses at Camiac and La Chauverie (SW France). J Taphon. 2012;10: 291–316.

[45] Marean CW. A critique of the evidence for scavenging by Neanderthals and early modern humans: new data from Kobeh Cave (Zagros Mountains, Iran) and Die Kelders Cave 1 layer 10 (South Africa). J Hum Evol. 1998;35: 111–136. 9719992 10.1006/jhev.1998.0224

[46] Henshilwood CS, D’Errico F, van Niekerk KL, Coquinot Y, Jacobs Z, Lauritzen S-E, et al A 100,000-year-old ochre-processing workshop at Blombos Cave, South Africa. Science. 2011;334: 219–222. 10.1126/science.1211535 21998386

[47] Vanhaeren M, d’Errico F, van Niekerk KL, Henshilwood CS, Erasmus RM. Thinking strings: Additional evidence for personal ornament use in the Middle Stone Age at Blombos Cave, South Africa. J Hum Evol. 2013;64: 500–517. 10.1016/j.jhevol.2013.02.001 23498114

[48] Archer W, Gunz P, van Niekerk KL, Henshilwood CS, McPherron SP. Diachronic Change within the Still Bay at Blombos Cave, South Africa. PLoS ONE. 2015;10: e0132428 10.1371/journal.pone.0132428 26134976 PMC4489860

[49] Nel TH. Micromammals, climate change and human behaviour in the Middle Stone Age, southern Cape, South Africa. PhD dissertation, University of Bergen. 2013.

[50] Jerardino A. The Problem with Density Values in Archaeological Analysis: A Case Study from Tortoise Cave, Western Cape, South Africa. South African Archaeol Bull. 1995;50: 21–27.

[51] Villa P, Soressi M, Henshilwood CS, Mourre V. The Still Bay points of Blombos Cave (South Africa). J Archaeol Sci. 2009;36: 441–460.

[52] Gravina B, Discamps E. MTA-B or not to be? Recycled bifaces and shifting hunting strategies at Le Moustier and their implication for the late Middle Palaeolithic in southwestern France. J Hum Evol. 2015;84: 83–98. 10.1016/j.jhevol.2015.04.005 25976251

